# Effects of Anosognosia on Perceived Stress and Cortisol Levels in Alzheimer's Disease

**DOI:** 10.1155/2012/209570

**Published:** 2012-03-01

**Authors:** Genevieve Arsenault-Lapierre, Victor Whitehead, Sonia Lupien, Howard Chertkow

**Affiliations:** ^1^Bloomfield Center for Research in Aging, Lady Davis Institute for Medical Research, Jewish General Hospital, Montreal, QC, Canada H3T 1E2; ^2^Center for Studies on Human Stress, Fernand-Seguin Research Center, Louis-H. Lafontaine Hospital, Montreal, QC, Canada H1N 3V2

## Abstract

Anosognosia, or unawareness of one's own cognitive deficits, may cause issues when measuring perceived stress and cortisol levels in Alzheimer's disease (AD) and Mild Cognitive Impairment (MCI). The goal of this study was to examine the effects of anosognosia on perceived stress and salivary cortisol levels in normal elderly (NE) adults, MCI individuals, newly diagnosed AD patients, and long-lasting AD patients, suspected to show more anosognosia. An anosognosia index for perceived stress was computed by subtracting the score on the Perceived Stress Scale measured in the participants and their relative. Cortisol levels were measured four times a day over two nonconsecutive days. Greater anosognosia for dementia correlated with greater anosognosia for perceived stress in the group as a whole. However, no correlation between cortisol levels and either anosognosia for dementia or perceived stress was observed. Our results suggest that measuring perceived stress in AD patients may be influenced by anosognosia.

## 1. Introduction

Cortisol is a hormone secreted when one faces a physical or a psychological stressor. As such, cortisol is conceived as a physiological marker of stress. When a stressor is perceived, the hypothalamo-pituitary-adrenal (HPA) axis is activated, the end product of which is the secretion of cortisol from the adrenal glands. Four psychological determinants have been shown to lead to the activation of the HPA axis and the secretion of cortisol: novelty, unpredictability, threat to self, and sense of loss of control [1]. Not everyone is sensitive to the same extent to each of these factors, but essentially, the more there are of these factors in any given situation, the more likely one will perceive the situation as stressful and the greater will be the secretion of cortisol [2].

Living with a diagnosis of Alzheimer's disease (AD) is especially stressful for the patients and their family and encompasses many, if not all, of the psychological determinants of a stress response. Indeed, in a review of qualitative studies on the impact of living with early dementia, Steeman and colleagues [3] reported that the “memory losses often threatens the patients' security, autonomy, and sense of being a meaningful member of society.” The authors added that the memory losses interfering with coping strategies may cause “frustration, uncertainty, and fear.” Similar conclusions were derived from a study by Clare and colleagues [4]. One would think that receiving the diagnosis of AD would trigger such a stress response. However, a group headed by Carpenter and colleagues [5] found that disclosure of AD diagnosis did not elicit significant increases on the Geriatric Depression Scale nor on the 20-item state version of the State-Trait Anxiety Inventory.

One important distinction in the field of human stress research is that to be stressful, a situation has to be perceived as such [6]. This brings us to an important limitation in the measurement of stress in AD, namely, the question as to whether AD patients are able to appraise their own level of stress. Patients with AD, and to some extent individuals with Mild Cognitive Impairment (MCI), often display anosognosia, or unawareness of their cognitive deficits [7–9]. The more the disease progresses, the more severe the anosognosia of the patients is [10]. It is therefore possible that AD patients and MCI individuals, unable to acknowledge their cognitive deficits, may also be unable to appraise their own stress.

Measures of perceived stress such as Cohen's 10-item Perceived Stress Scale (PSS-10) [11] have been used in the normal elderly (NE) population, and norms have been already established [12]. To our knowledge only one study has examined PSS-10 in AD patients. Wahbeh and colleagues [13] found that AD patients and their caregivers did not show significantly higher perceived stress compared to older adults. Consequently, norms have yet to be established for AD patients and individuals with MCI.

Higher cortisol levels have been found to correlate with increased perceived stress in some studies examining various populations [14, 15] but not in other studies [16–18]. Wahbeh and colleagues [13] have measured the association between cortisol levels and perceived stress in AD patients, and found a positive trend between cortisol levels measured at 30 minutes after awakening and the patients' scores on PSS-10. Although this result suggests that AD patients are able to appraise stressors with the concomitant increase of cortisol levels that is associated with this appraisal, it is not clear at this point in the literature whether the apparition of anosognosia that develops with the disease eliminates the association between perceived stress and cortisol levels in these populations. It is possible that with the progression of the disease over time, more anosognosia surfaces, counteracting the expected increase in cortisol levels of increasingly more advanced, and less insightful, AD patients.

There are many scales that measure anosognosia, such as the Anosognosia Questionnaire-Dementia (ANO) [19]. Like in most scales assessing anosognosia, the discrepancy between the scores of the participants and those of their relatives on a list of cognitive deficits items is used as an index of anosognosia for dementia. It would be interesting to look at a similar anosognosia index for perceived stress. To our knowledge, this has not been done or published in the literature before.

The goal of this study was to examine the effects of anosognosia on the psychological and physiological markers of stress in Alzheimer's disease. We wanted to examine this in a full cognitive spectrum of older adults, including healthy NE who have no anosognosia for dementia, MCI individuals who are expected to have lower levels of anosognosia for dementia, newly diagnosed AD patients, and AD patients who had the diagnosis for a longer period, thus suspected to show more anosognosia. We hypothesized that anosognosia for dementia should correlate positively with anosognosia for perceived stress and inversely correlate with cortisol levels.

## 2. Methods

### 2.1. Subjects

A convenience sample of 20 MCI individuals and 29 AD patients was recruited at the memory clinic of the Jewish General Hospital, Montreal, Canada. In addition, 20 normal elderly (NE) subjects without memory loss were recruited from the general population. Each participant underwent a battery of neuropsychological tests carried out by neuropsychologists or trained research assistants. CT scans were performed to rule out any organic cause of cognitive dysfunction. A diagnosis of MCI or AD was made by consensus at clinical meetings composed of neurologists, nurses, geriatricians, and neuropsychologists who attended to the patients.

Clinical criteria for MCI were those proposed by Petersen and colleagues [20]: (1) having subjective memory complaints, (2) small but measurable deficits (usually at least 1 to 1.5 standard deviations below norms) on neuropsychological tests adjusted for age and education, (3) lack of significant functional or social impairment, and (4) not meeting criteria for dementia. For a diagnosis of AD, the clinical criteria were those of the NINCDS-ADRDS [21]. We considered a participant to have newly diagnosed AD, labeled “new AD,” if the diagnosis occurred within the past six months of testing; consequently, participants who have had AD for longer than six months were considered as having long-standing AD, hereafter labeled “old AD.” The distinction from new AD and old AD is based on the fact that old AD patients are hypothesized to have higher anosognosia for dementia, and possibly for perceived stress, as well as to have more time to adjust to a new diagnosis of AD. Exclusion criteria were the presence of other organic or psychiatric disorder that could account for the cognitive deficits in MCI and AD patients, and the presence of any cognitive deficits in NE. 

Each participant had to designate an individual in their immediate social circle (a friend, a spouse, a child, or a caregiver, hereafter referred to as a relative) to participate in the study. Medical charts reviews were carried out to determine the participants' medications that could affect cortisol and perceived stress ratings. Informed consent was received from every participant and their relative, and the protocol was approved by Research Ethic Board of the Jewish General Hospital, Montreal, Canada.

### 2.2. Anosognosia Index for Dementia (ANO)

The Anosognosia Questionnaire-dementia (ANO), [19] was given to each participant. This questionnaire has been validated and consists of 30 items assessing one's memory, daily life, and behavioral and psychological symptoms on four-point scales. Both the participant's version and the relative's version of the questionnaire asked the same questions about the participant; the difference in the scores between these versions was then used as an index of anosognosia for dementia. The greater is the difference in the score of the AD patient and the relative on the scale, the greater is the anosognosia index assigned to the AD patient.

### 2.3. Anosognosia Index for Perceived Stress (PSS)

The 10-item Perceived Stress Scale (PSS-10) [11] was administered to every participant and their relative. It constitutes one of the most widely used quantitative questionnaires on perceived stress and measures the stress perceived by an adult over the previous month. An index of anosognosia for perceived stress was devised by subtracting the score of the participant from that of their relative, in a manner similar to how we obtained the index of anosognosia for dementia: PSS = PSS-10_relative_  − PSS-10_participant_. About half of the participants and their relatives were given an incomplete version of the PSS-10 on the first visit (measurement error), resulting in two missing items for about 40% of the study sample, making comparisons with norms difficult. An adjusted score out of 10 was computed from the score out of 8 for every subject.

### 2.4. Salivary Cortisol Levels

Participants were instructed to collect four samples of saliva per day on two nonconsecutive days. The four samples were taken upon awakening, 30 minutes after awakening, at 2PM, and at bedtime and comprised passive drool collected in plastic tubes. The participants and/or caregivers were then instructed to write down the exact time, report any unusual event that might have occurred before the saliva collection, and rate the mood of the participant on a log sheet. We asked the participants and/or their caregivers to subsequently store the saliva samples in their freezer to prevent degradation until they could be collected for analysis. Radioimmunoassays were performed at the Douglas Institute of Mental Health, Montreal, Canada. Saliva measured for the four collection times were averaged across the two days, controlling for the day-to-day variation in cortisol secretion.

### 2.5. Procedure

The participants' and relatives' versions of both the anosognosia and the PSS-10 questionnaires were administered at the first home visit, along with the instructions for the salivary collection. About two weeks after the first visit, a second appointment was scheduled to pick up the saliva samples. At the same time the Mini-Mental State Evaluation (MMSE) [22] and Montreal Cognitive Assessment (MoCA) [23] were given to measure the cognitive function of the participants.

### 2.6. Statistical Analyses

Multiple analyses of variance (ANOVAs) and Pearson's correlations were carried out using SPSS version 16.0 (SPSS Inc., Chicago, IL).

## 3. Results

From November 2009 to January 2010, a total of 22 NE, 21 MCI individuals, 12 new AD patients (who received a diagnosis of AD within the past six months), and 16 old AD patients (who received a diagnosis of AD earlier than the past six months) and their relatives agreed to participate in the study. There were no differences in age, education, or gender between the participants included and the initial bigger cohort, nor between the participants for whom we have a perceived stress score out of 10 and those for whom we have a perceived stress score out of 8. We found very strong correlations between scores out of 10 and scores out of 8 for the participants on whom we had complete PSS-10 questionnaires (*r* = 0.98, *P* < 0.01, *n* = 43), suggesting that the two missing items for half of the sample had no major impact, allowing us to perform the intended analyses. One old AD participant was excluded from the analyses due to extreme levels of cortisol (more than 3 standard deviations above the mean for old AD group). A review of concomitant medications amongst participants revealed that more MCIs were on medication for diabetes than the other groups (*n*_NE_ = 0; *n*_MCI_ = 5; *n*_newAD_ = 0; *n*_oldAD_ = 0), whereas more AD patients (both new and old) were on sedatives/antidepressants (*n*_NE_ = 1; *n*_MCI_ = 1; *n*_newAD_ = 4; *n*_oldAD_ = 5) and acetyl cholinesterase inhibitors (*n*_NE_ = 0; *n*_MCI_ = 0; *n*_newAD_ = 6; *n*_oldAD_ = 8). There were no differences in demographics, anosognosia for dementia, anosognosia for perceived stress, or cortisol between the MCI who were on diabetic medications and those who were not. Similarly, there were no statistical difference in demographics, anosognosia for dementia, PSS-10, anosognosia for perceived stress, or cortisol between the AD who were on sedatives, antidepressants, and/or acetyl cholinesterase inhibitor and those who were not.

The participants in the four groups were similar in age and education, but there were more women participants in the NE group compared to the other groups (*X*_3_^2^ = 8.8, *P* = 0.03). As expected, the groups differed in MMSE and MoCA scores (all *P* < 0.01). Demographic information is presented in Table 1.

There were no differences across the diagnostic groups in the level of perceived stress (PSS-10) as reported by the participants (*F*_(3,70)_ = 2.5, *P* = 0.07). There was a trend, however, mainly driven by MCI who reported higher perceived stress than the other groups (not significant). However, there were significant diagnostic group differences in the level of perceived stress (PSS-10) as reported by the relatives (*F*_(3,70)_ = 7.3, *P* < 0.01). Posthoc analyses showed that the relatives reported less stress for the NE participants than for both new and old AD participants. In addition, the relatives reported less stress for the MCI participants than the old AD participants (all *P* < 0.05).

**Figure 1:**
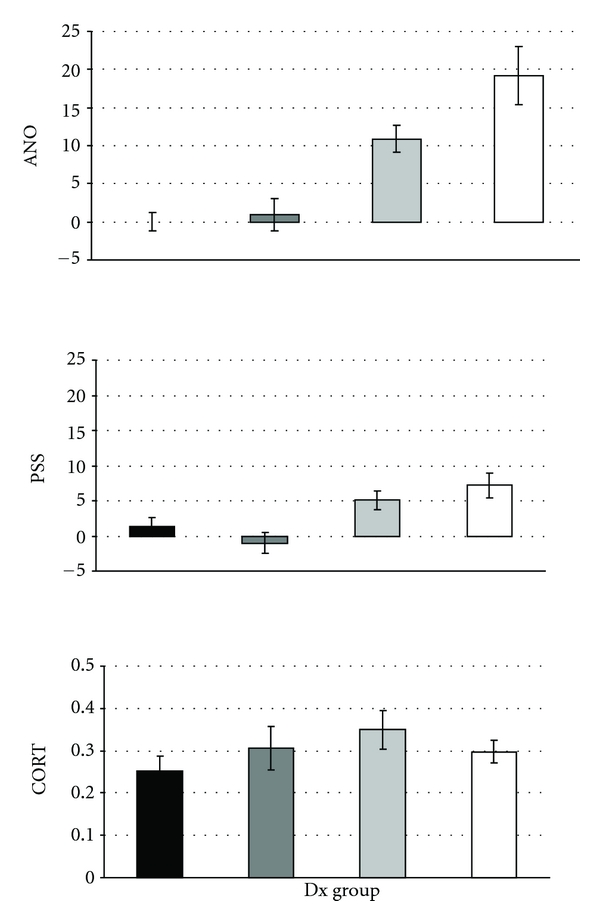
Scores on anosognosia for dementia, anosognosia for perceived stress, and cortisol levels in normal elderly, individuals with Mild Cognitive Impairment, newly diagnosed, and long-lasting Alzheimer's disease patients. These graphs represent the scores on anosognosia for dementia (ANO), anosognosia for perceived stress (PSS), and cortisol levels (CORT, in *μ*g/dL) in 21 normal elderly (NE, black bars), 20 individuals with Mild Cognitive Impairment (MCI, dark grey bars), 12 newly diagnosed (new AD, light grey bars), and 17 long-lasting (old AD, white bars) Alzheimer's disease patients.

The ANOVAs revealed statistically significant group differences in the anosognosia index for dementia (ANO, *F*_(3,  67)_ = 15.3, *P* < 0.01). Post-hoc analyses, using Bonferroni's corrections, showed that the NE had lower anosognosia for dementia (ANO) than new AD (*P* = 0.02) and old AD (*P* < 0.01) and that, similarly, the MCI had lower anosognosia for dementia than new AD (*P* = 0.04) and old AD (*P* < 0.01). The ANOVAs revealed statistically significant group differences in the anosognosia for perceived stress (PSS, *F*_(3,  67)_ = 6.15, *P* < 0.01). Post-hoc analyses also revealed that NE (*P* = 0.03) and MCI (*P* = 0.01) had lower anosognosia for stress (PSS) than old AD. There were no group difference for cortisol level (*F*_(3,  63)_ = 0.77, *P* = 0.51). These results are presented in Figure 1.

**Figure 2:**
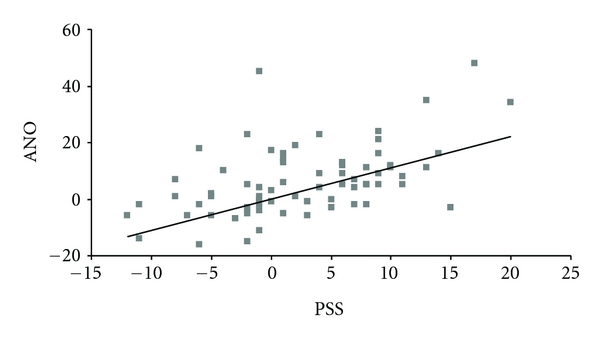
Relationship between anosognosia for dementia and anosognosia for perceived stress in normal elderly, individuals with Mild Cognitive Impairment, and Alzheimer's disease patients. This graph represents the lack of association between the scores on anosognosia for dementia (ANO) and on anosognosia for perceived stress (PSS) in all the subjects. PSS is calculated as a difference score on the PSS-10 as reported by the participants themselves versus the PSS-10 as reported by their relatives.

To evaluate the association between the indices of anosognosia for dementia (ANO) and anosognosia for perceived stress (PSS), Pearson's correlations were carried out. We found a statistically significant positive correlation between the two scales across the groups (*r* = 051, *P* < 0.01, *n* = 71; see Figure 2) showing that greater anosognosia for dementia were associated with greater anosognosia for perceived stress. Carrying these analyses in the diagnostic groups independently, we found a positive correlation between the two scales in the NE only (*r* = 0.98, *P* < 0.01, *n* = 22). When we combined new and old AD together, we also found a positive correlation between the two indices of anosognosia (*r* = 0.39, *P* = 0.04, *n* = 29).

To evaluate the association between cortisol levels and the anosognosia indices for dementia and perceived stress, we performed Pearson's correlations across the diagnostic groups. We did not find any statistically significant correlations between cortisol levels and either the anosognosia index for dementia (ANO, *r* = 0.10, *P* = 0.42, *n* = 64) or the anosognosia index for perceived stress (PSS, *r* = −0.11, *P* = 41, *n* = 64).

## 4. Discussion

This study investigated whether a lack of insight of one's memory deficits correlates with one's lack of insight for perceived stress. Using targeted questionnaires, we found that an index of anosognosia for dementia correlated significantly with an index of anosognosia for perceived stress showing that measuring perceived stress in AD, and possibly in MCI individuals, may cause some problems due to the inability of AD patients and some MCI individuals to acknowledge their cognitive state.

Indeed, we found that AD patients, and more precisely AD patients who have been diagnosed for a longer period of time, displayed more anosognosia for perceived stress than NE or MCI individuals. Similarly, both newly diagnosed and long-standing AD patients also showed greater anosognosia for dementia than either NE or MCI individuals. These results are similar to findings in the literature, where AD patients showed anosognosia for dementia [7–9], but not MCI individuals [24–27]. Although the difference between new AD and old AD was not significant in the anosognosia for dementia scale, our results indicated that with the disease progression and time, the severity of anosognosia for perceived stress, and to some extend for dementia, increased.

Surprisingly, we did not find group differences in cortisol levels. This contradicts numerous previous studies (including our own) that found differences between the cortisol levels of NE and AD groups [28–32], and between NE and MCI groups [32, 33]. The small sample size of this study, paired with the high variability of cortisol measurements across the days and seasons [32], almost certainly explains the failure to find statistically significant group differences.

Another goal for this study was to examine the relationship between anosognosia for dementia and perceived stress with physiological marker of stress, namely, cortisol levels. We did not find an association between cortisol levels and anosognosia for dementia, suggesting that anosognosia for dementia does not affect the levels of cortisol secreted by the participants. Interestingly, we did not find an association between cortisol levels and anosognosia for perceived stress. This adds to the body of literature that found no association of cortisol and perceived stress in various populations [16–18]. However, we did not find the expected group differences in cortisol secretion with progression of the disease, preventing us from driving more in-depth conclusions on the association, or lack thereof, between cortisol and anosognosia for dementia or perceived stress.

Beside the small sample size, a few limitations need to be addressed when interpreting our results. First, there were more women in the NE group compared to the other groups, especially when compared to AD patients. There exist gender differences in stress [34, 35], and there exist gender by age interaction effects on cortisol levels in response to a stressor [36]. Second, it is worth noting that more MCIs were treated for diabetes, and diabetic individuals have been found to secrete higher cortisol levels [37–39]. Third, and perhaps most importantly, more AD patients (both new and old) were on sedatives or antidepressants. These medications may affect the level of perceived stress and thus explain the relative anosognosia for perceived stress measured in these patients. The limited number of participants with up-to-date medication information, especially in the NE, prevented us from further conclusions regarding the effect of medications in this study. Finally, a subsample of the participants included in this study had incomplete PSS-10 questionnaires, where two items were missing. Although there was high correlation between the complete version and the incomplete versions in the individuals for which we had complete data, it remains difficult to assess whether the participants included in our study had scores within the norms for older adults as suggested by Cohen and Williamson [12]. When scores were adjusted to a total of 10, the means of the four groups (see Table 1) were within the suggested normal range (mean: 12.0 ± standard deviation: 6.3).

## 5. Conclusion

Despite these shortcomings, this study suggests that anosognosia for dementia has an impact on psychological markers of stress, but not on physiological markers of stress in Alzheimer's disease. It is necessary to replicate these findings in a larger sample while controlling for gender and, medications. However our results suggest that measuring perceived stress in AD patients may be influenced by the extent of anosognosia and consequently, caution should be taken when assessing association between perceived stress and cortisol levels in these populations.

## Figures and Tables

**Table 1:** Demographic information for the normal elderly, individuals with Mild Cognitive Impairement, newly diagnosed, and long-lasting Alzheimer's disease patients.

	NE	MCI	New AD	Old AD
Age	77.7 (1.3)	77.1 (1.3)	80.0 (0.8)	77.9 (1.4)
Education	15.2 (0.6)	15.9 (1.0)	13.1 (1.6)	13.9 (1.3)
Gender (M : W)*	9 : 13	14 : 7	7 : 5	14 : 2
MMSE*	28.7 (1.4)	27.8 (2.0)	26.0 (1.9)	20.1 (5.9)
MoCA*	27.2 (0.6)	23.5 (0.7)	19.1 (1.7)	14.8 (1.4)
PSS-10 adjusted				
* Participant*	7.6 (0.92)	12.6 (1.4)	8.8 (1.7)	10.2 (8.0)
Relative*	9.3 (1.5)	11.3 (6.3)	15.1 (7.4)	19.2 (6.7)
ANO*	−0.5 (1.2)	1.0 (2.1)	10.9 (1.7)	19.2 (3.8)

This table represents the mean (standard error of the mean) and men to women ratio (M : W) for the 22 normal elderly (NE), 21 individuals with Mild Cognitive Impairment (MCI), 12 newly diagnosed Alzheimer's disease patients (new AD), and 17 long-lasting Alzheimer's disease patients (old AD). Age and education are in years. MMSE stands for Mini-Mental State Evaluation; MoCA stands for Montreal Cognitive Assessment, PSS-10 adjusted stands for 10-item Perceived Stress Scale out of 8, adjusted to a score of 10, as reported by the participants themselves, and as reported by their relatives; ANO stands for anosognosia for dementia score. *Denotes a significant group difference with a *P*-value less than 0.05.
